# Supplementary material for: Divergent Influenza-Like Viruses of Amphibians and Fish Support an Ancient Evolutionary Association
Source: Viruses. 2020 Sep 18;12(9):1042. doi: 10.3390/v12091042 (PMC7551885; doi:10.3390/v12091042)
Supplement: Supplementary file 1 [file viruses-12-01042-s001.zip › viruses-930212-supplementary.pdf]

viruses

Supplementary

Divergent Influenza-Like Viruses of Amphibians and Fish Support an Ancient Evolutionary Association

Rhys Parry [1,*,†], Michelle Wille [2,*,†], Olivia M. H. Turnbull [3], Jemma L. Geoghegan [4,5] and Edward C. Holmes [2,*]

1 Australian Infectious Diseases Research Centre, School of Chemistry and Molecular Biosciences, The University of Queensland, Brisbane, QLD 4067, Australia
2 Marie Bashir Institute for Infectious Diseases and Biosecurity, School of Life & Environmental Sciences and School of Medical Sciences, The University of Sydney, Sydney, NSW 2006, Australia
3 Department of Biological Sciences, Macquarie University, Sydney, NSW 2109, Australia; olivia.turnbull@hdr.mq.edu.au
4 Department of Microbiology and Immunology, University of Otago, Dunedin 9016, New Zealand; jemma.geoghegan@otago.ac.nz
5 Institute of Environmental Science and Research, Wellington 5018, New Zealand
* Correspondence: r.parry@uq.edu.au (R.P.); michelle.wille@sydney.edu.au (M.W.); edward.holmes@sydney.edu.au (E.C.H.); Tel.: +617-3365-3302 (R.P.); +61-2-9114-2199 (M.W.); +61-2-9351-5591 (E.C.H.)
† These authors contributed equally to this work.

Abstract: Influenza viruses (family *Orthomyxoviridae*) infect a variety of vertebrates, including birds, humans, and other mammals. Recent metatranscriptomic studies have uncovered divergent influenza viruses in amphibians, fish and jawless vertebrates, suggesting that these viruses may be widely distributed. We sought to identify additional vertebrate influenza-like viruses through the analysis of publicly available RNA sequencing data. Accordingly, by data mining, we identified the complete coding segments of five divergent vertebrate influenza-like viruses. Three fell as sister lineages to influenza B virus: salamander influenza-like virus in Mexican walking fish (*Ambystoma mexicanum)* and plateau tiger salamander (*Ambystoma velasci*), Siamese algae-eater influenza-like virus in Siamese algae-eater fish (*Gyrinocheilus aymonieri*) and chum salmon influenza-like virus in chum salmon (*Oncorhynchus keta*). Similarly, we identified two influenza-like viruses of amphibians that fell as sister lineages to influenza D virus: cane toad influenza-like virus and the ornate chorus frog influenza-like virus, in the cane toad (*Rhinella marina)* and ornate chorus frog (*Microhyla fissipes)*, respectively. Despite their divergent phylogenetic positions, these viruses retained segment conservation and splicing consistent with transcriptional regulation in influenza B and influenza D viruses, and were detected in respiratory tissues. These data suggest that influenza viruses have been associated with vertebrates for their entire evolutionary history.

Keywords: *Orthomyxoviridae*; influenza; metatranscriptomics; fish; amphibians; evolution; phylogeny

Table S1. Protein domain families found in influenza viruses.

Protein domain families	Accession	Description
Flu_PB1	pfam00602	Influenza RNA-dependent RNA polymerase subunit PB1; Two GTP binding sites exist in this protein.
Flu_PB2 super family	cl20020	Influenza RNA-dependent RNA polymerase subunit PB2; PB2 can bind 5' end cap structure of RNA.
Flu_PA super family	cl02905	Influenza RNA-dependent RNA polymerase subunit PA.
Hemagglutinin super family	cl10565	Haemagglutinin; Haemagglutinin from influenza virus causes membrane fusion of the viral membrane with the host membrane. Fusion occurs after the host cell internalizes the virus by endocytosis. The drop of pH causes release of a hydrophobic fusion peptide and a large conformational change leading to membrane fusion.
Sialidase super family	cl21531	Sialidases /neuraminidases function to bind and hydrolyze terminal sialic acid residues from various glycoconjugates as well as playing roles in pathogenesis, bacterial nutrition and cellular interactions. They have a six-bladed beta-propeller fold. This hierarchy includes eubacterial, eukaryotic, and viral sialidases.
Hema_esterase super family	cl28016	Hemagglutinin esterase;
Hema_stalk super family	cl07368	Influenza C hemagglutinin stalk; This domain corresponds to the stalk segment of hemagglutinin in influenza C virus. It forms a coiled coil structure.
Neur super family	cl19749	Neuraminidase; Neuraminidases cleave sialic acid residues from glycoproteins. Belong to the sialidase family - but this alignment does not generalize to the other sialidases. Structure is a 6-sheet beta propeller.
NB super family	cl04425	NB glycoprotein; The NB glycoprotein is found in Influenza type B virus. Its function is unknown.
Flu_M1	pfam00598	Influenza Matrix protein (M1); This protein forms a continuous shell on the inner side of the lipid bilayer.
Flu_M1_C super family	cl27878	Influenza Matrix protein (M1) C-terminal domain; This region is thought to be a second domain of the M1 matrix protein.
Flu_B_M2 super family	cl04739	Influenza B matrix protein 2 (BM2); M2 is synthesized in the late phase of infection and incorporated into the virion. It may be phosphorylated in vivo. The function of BM2 is unknown.
CM1 super family	cl03846	Influenza C virus M1 protein; This family represents the matrix 1 protein of influenza C virus. The protein is the product of a spliced mRNA. Small quantities of the unspliced mRNA are found in the cell additionally encoding the M2 protein (see pfam03021).
CM2 super family	cl03843	Influenza C virus M2 protein; Influenza C virus M1 protein is encoded by a spliced mRNA. The unspliced mRNA is also found in small quantities and can encode the protein represented by this family.
Flu_B_NS1 super family	cl03792	Influenza B non-structural protein (NS1); A specific region of the influenza B virus NS1 protein, which includes part of its effector domain, blocks the covalent linkage of ISG15 to its target proteins both in vitro and in infected cells. Of the several hundred proteins induced by interferon (IFN) alpha/beta, the ubiquitin-like ISG15 protein is one of the most predominant. Influenza A virus employs a different strategy: its NS1 protein does not bind the ISG15 protein, but little or no ISG15 protein is produced during infection.
Flu_C_NS1 super family	cl04118	Influenza C non-structural protein (NS1); The influenza C virus genome consists of seven single-stranded RNA segments. The shortest RNA segment encodes a 286 amino acid non-structural protein NS1. This protein contains 6 conserved cysteines that may be functionally important, perhaps binding to a metal ion.
Flu_C_NS2 super family	cl04153	Influenza C non-structural protein (NS2); The influenza C virus genome consists of seven single-stranded RNA segments. The shortest RNA segment encodes a 286 amino acid non-structural protein NS1 pfam03506 as well as the NS2 protein. The NS2 protein is only about 60 amino acids in length and of unknown function.

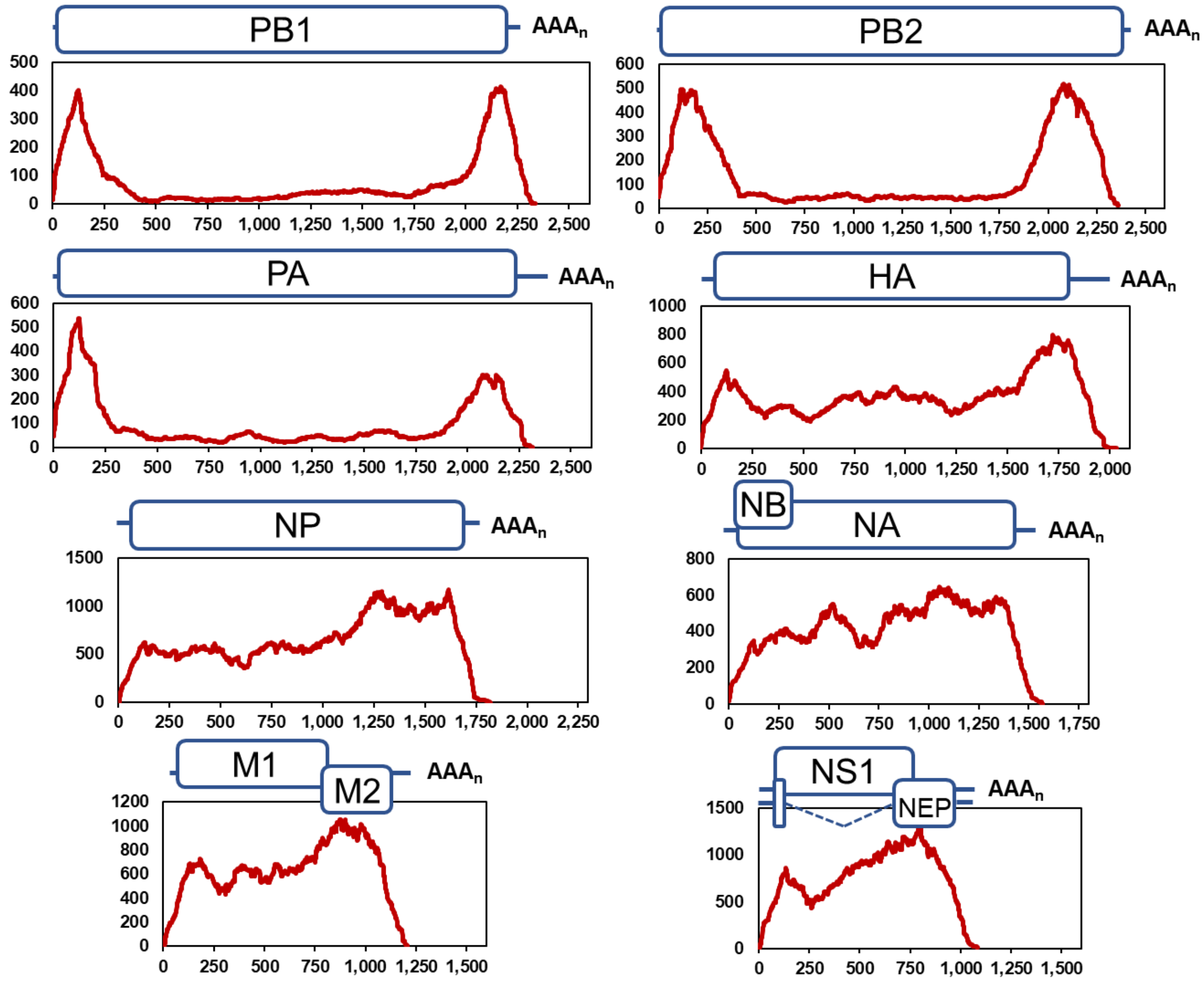

Figure S1. Genome organization and mapping coverage of Salamander influenza-like virus. Coloured boxes correspond to predicted protein domains. PB1, RNA-dependent RNA polymerase basic subunit 1; PB2, RNA-dependent RNA polymerase basic subunit 2; PA, RNA-dependent RNA polymerase acidic subunit; NP, nucleoprotein; HA, hemagglutinin; NA, neuraminidase; M1/M2, matrix proteins 1 & 2; NS1, non-structural protein; NEP, nuclear export protein. Isoform variants of genes indicated by dotted lines. Sequencing data taken from spinal cord samples of *Ambystoma mexicanum* (BioProject Accession: PRJNA506973, Pooled samples: SRR8244387, SRR8244388, SRR8244389) [1].

Table S2. Annotation of Salamander influenza-like virus.

	Location; Amino acid size	Closest BLASTp hit; Query cover (%); Identity (%)	*E*-value	Protein domain families	Accession	Interval	*E*-value
PB1 Segment 2331nt							
ORF1 PB1	23-2272; 749aa	polymerase PB1 [Influenza B virus (B/Victoria/593/2011)] AGX21686.1; 100%; 75.57%	0	Flu_PB1	pfam0060	21–740	0
PB2 Segment 2362nt							
ORF1 PB2	23-2329; 769aa	polymerase PB2 [Influenza B virus] AZY32600.1; 99%; 61.83%	0	Flu_PB2 super family	cl20020	13–761	0
PA Segment 1570nt							
ORF1 PA	33-2231; 732aa	polymerase PA [Influenza B virus (B/Indiana/07/2016)] ANW74127.1; 97%; 59.64%	0	Flu_PA super family	cl02905	21–715	0
HA Segment 1981nt							
ORF1 HA	67-1830; 587aa	hemagglutinin [Wuhan spiny eel influenza virus] AVM87624.1; 95%; 43.34%	8×10^{-161}	Hemagglutinin super family	cl10565	26–587	8.38×10^{-113}
NP Segment 1809nt							
ORF1 NP	78-1718; 546aa	nucleoprotein [Influenza B virus (B/Malaysia/10928/1996)] AFJ80155.1; 89%; 50.60%	1×10^{-171}	Flu_NP super family	cl27387	48–541	0
NA Segment 1555nt							
ORF1 NA	68-1471; 467aa	neuraminidase [Influenza B virus] AVP05457.1; 94%; 46.29%	5×10^{-146}	Neur super family	cl19749	118–448	3.14×10^{-111}
ORF2 NB	61-330; 89aa	NB protein [Influenza B virus (B/California/02/2017)] AQS20108.1; 65%; 35.00%	0.023	NB super family	cl04425	19–59	0.17
M Segment 1201nt							
ORF1	45-791; 248aa	matrix protein 1 [Influenza B virus (B/California/07/2015)] ANE25075.1; 99%; 48.79%	2×10^{-82}	Flu_M1<br>Flu_M1_C super family	pfam00598<br>cl27878	2–155<br>158–247	2.71×10^{-44}<br>9.22×10^{-13}
ORF2	788-1138; 116aa	BM2 protein [Influenza B virus (B/Florida/52/2016)] ANW76071.1; 93%; 25.69%	0.33	Flu_B_M2 super family	cl04739	6–114	1.04×10^{-3}
NS Segment Isoform 1: 1083nt							
ORF1	70-795; 242aa	nonstructural protein 1 [Influenza B virus] QCG85998.1; 94%; 29.01%	1×10^{-25}	Flu_B_NS1 super family	cl03792	5-216	2.78×10^{-26}
ORF2	683-1003; 107aa	nuclear export protein [Influenza B virus] AXA17238.1; 95%; 25.71%	0.012				
NS Segment Isoform 2: 512nt Splice junction 70–96; 668–1003 of NS Segment Isoform 1 reference							
NEP	120aa	nuclear export protein [Influenza B virus (B/Wisconsin/46/2017)] ATP82168.1; 91%; 26.55%	1×10^{-4}				

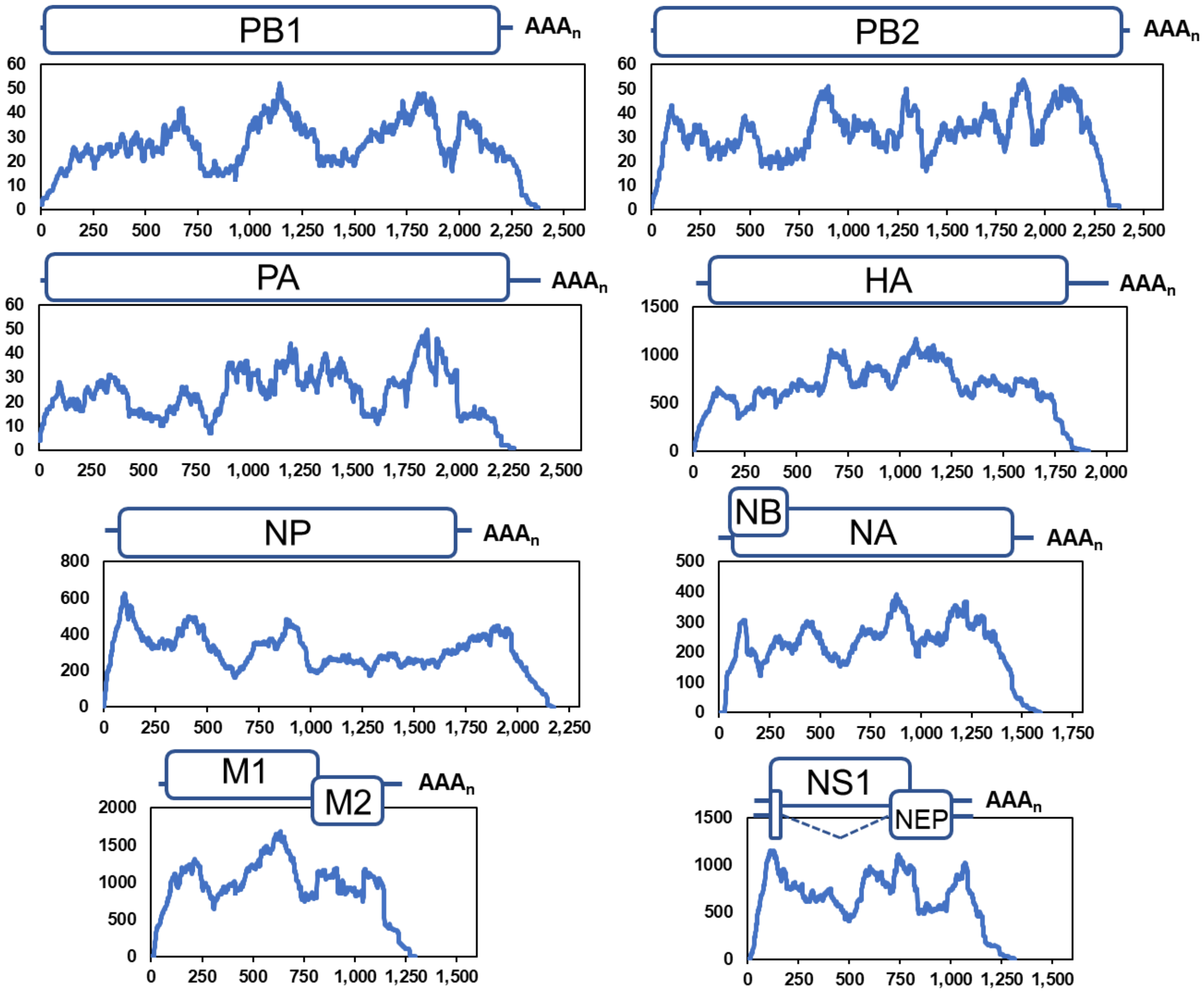

Figure S2. Genome organization and mapping coverage of Chum salmon influenza-like virus. Coloured boxes correspond to predicted protein domains. PB1, RNA-dependent RNA polymerase basic subunit 1; PB2, RNA-dependent RNA polymerase basic subunit 2; PA, RNA-dependent RNA polymerase acidic subunit; NP, nucleoprotein; HA, hemagglutinin; NA, neuraminidase; M1/M2, matrix proteins 1 & 2 ; NS1, non-structural protein; NEP, nuclear export protein. Isoform variants of genes indicated by dotted lines. Sequencing data taken from gill samples of *Oncorhynchus keta* (SRA Accession: SRR6998471) [2].

Table S3. Annotation of Chum salmon influenza-like virus.

	Location; Amino acid size	Closest BLASTp hit; Query cover (%); Identity (%)	*E*-value	Protein domain families	Accession	Interval	*E*-value
PB1 Segment 2378nt							
ORF1 PB1	50-2302; 749aa	polymerase PB1 [Influenza B virus] QHI05420.1 100%; 69.50%	0.0	Flu_PB1	pfam0060	1–739	0
PB2 Segment 2378nt							
ORF1 PB2	23-2317; 765aa	PB2 [Influenza B virus (B/Memphis/5/93)] AAU94860.1; 99%; 57.05%	0	Flu_PB2 super family	cl20020	3–758	0
PA Segment 2276nt							
ORF1 PA	43-2208; 720aa	polymerase PA [Influenza B virus (B/Taiwan/45/2007)] ACO06009.1; 99%; 51.88%	0.0	Flu_PA super family	cl02905	21–713	0
HA Segment 1914nt							
ORF1 HA	31-1767; 578aa	hemagglutinin [Influenza B virus (B/Palermo/109/2013)] AMB72263.1; 90%; 32.90%	2×10^{-75}	Hemagglutinin super family	cl10565	26–520	4.48×10^{-82}
NP Segment 2174nt							
ORF1 NP	82-2052; 656aa	nucleoprotein [Influenza B virus (B/NYMC BX-15(Lee/1940-Hawaii/33/2004))]; ACO05951.1; 82%; 44.50%	5×10^{-152}	Flu_NP super family	cl27387	129–652	3.16×10^{-156}
NA Segment 1580nt							
ORF1 NA	64-1458; 463aa	neuraminidase [Influenza B virus (B/Waikato/70/2005)] AGX22801.1; 97%; 38.76%	3×10^{-97}	Sialidase super family	cl21531	88–58	7.18×10^{-88}
ORF2 NB	84-512; 141aa						
M Segment 1313nt							
ORF1	30-770; 246aa	matrix protein 1 [Influenza B virus] AVR49412.1; 89%; 34.53%	1×10^{-45}	Flu_M1<br>Flu_M1_C super family	pfam00598<br>cl27878	9–153<br>158–242	9.51×10^{-32}<br>1.87×10^{-6}
ORF2	733-1116; 126aa	No BLASTp hit. Transmembrane domain predicted at N terminus.		uvrC super family	cl35109	49–110	7.14×10^{-4}
NS Segment Isoform 1: 1360nt							
ORF1	54-1055; 333aa	NS [Wuhan spiny eel influenza virus] AVM87628.1; 20%; 38.36%	1.8	Flu_B_NS1 super family	cl03792	4–51	0.05
ORF2	879-1214; 111aa						
NS Segment Isoform 2: 645nt, Splice junction 1–196:922–1360 of NS segment isoform 1 as reference							
ORF1	54-545; 163aa						

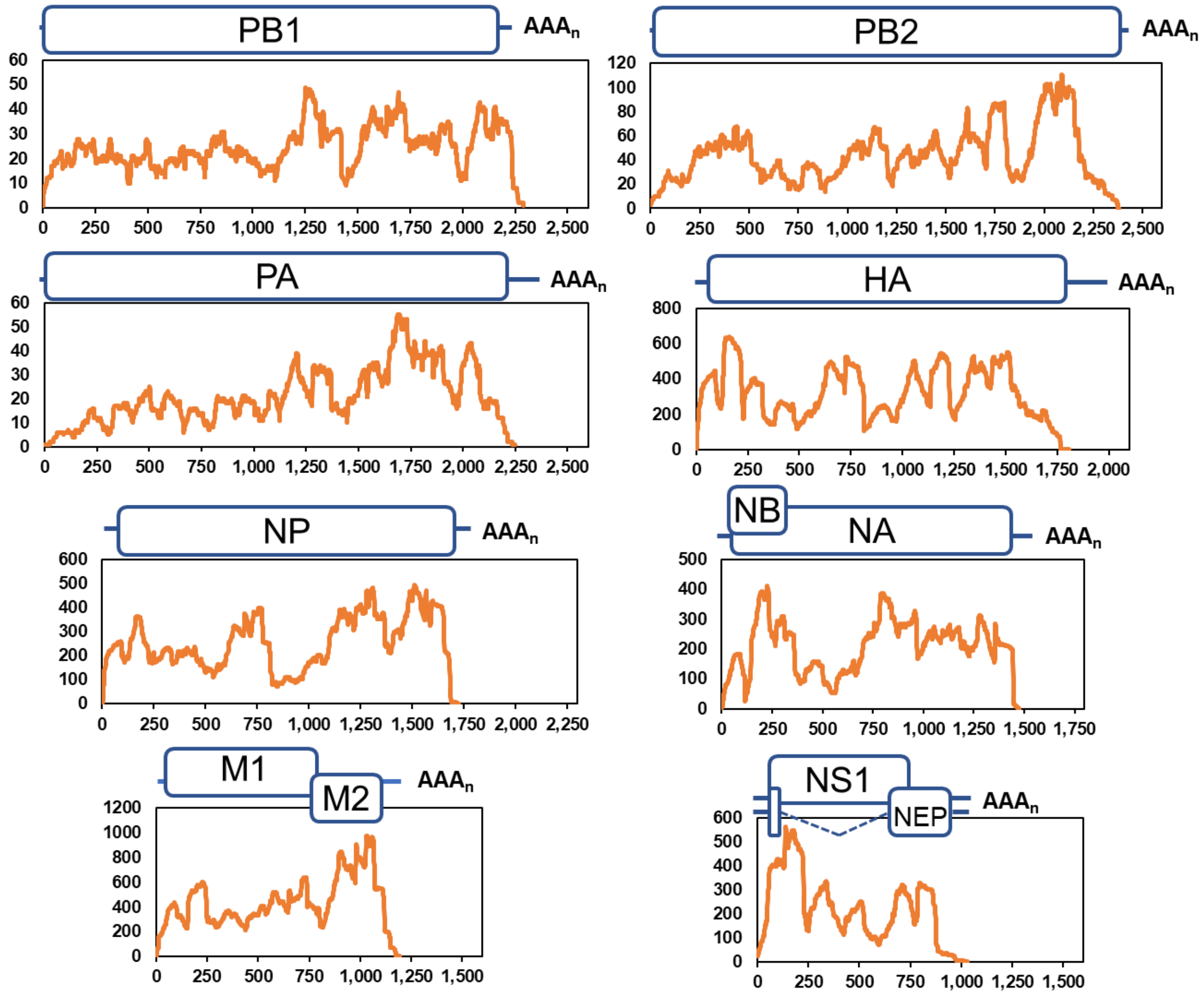

Figure S3. Genome organization and mapping coverage of Siamese algae-eater influenza-like virus. Coloured boxes correspond to predicted protein domains. PB1, RNA-dependent RNA polymerase basic subunit 1; PB2, RNA-dependent RNA polymerase basic subunit 2; PA, RNA-dependent RNA polymerase acidic subunit; NP, nucleoprotein; HA, hemagglutinin; NA, neuraminidase; M1/M2, matrix proteins 1 & 2; NS1, non-structural protein; NEP, nuclear export protein. Isoform variants of genes indicated by dotted lines. Sequencing data are taken from an individual library of gill samples sampled as part of the Fish-T1K Project (SRA Accession: SRR5997773) [3].

Table S4. Annotation of Siamese algae-eater influenza-like virus.

	Location; Amino acid size	Closest BLASTp hit; Query cover (%); Identity (%)	*E*-value	Protein domain families	Accession	Interval	*E*-value
PB1 Segment 2291nt							
ORF1 PB1	17-2290; 756aa	polymerase PB1 [Influenza B virus] QHI05420.1; 99%; 69.46%	0.0	Flu_PB1	pfam0060	6–747	0
PB2 Segment 2380nt							
ORF1 PB2	24-2360; 777aa	polymerase PB2 [Influenza B virus (B/New York/1121/2007)] AHL92298.1; 99%; 48.52%	0	Flu_PB2 super family	cl20020	3–769	0
PA Segment 2251nt							
ORF1 PA	20-2245; 740aa	PA [Wuhan spiny eel influenza virus] AVM87622.1; 96%; 53.89%	0.0	Flu_PA super family	cl02905	21–714	0
HA Segment 1807nt							
ORF1 HA	24-1754; 575aa	hemagglutinin [Wuhan spiny eel influenza virus] AVM87624.1; 97%; 33.84%	6×10^{-91}	Hemagglutinin super family	cl10565	20–576	5.21×10^{-103}
NP Segment 1725nt							
ORF1 NP	43-1671; 541aa	nucleocapsid protein [Influenza A virus] QDA17788.1; 88%; 44.12%	2×10^{-135}	Flu_NP super family	cl27387	36–542	1.68×10^{-158}
NA Segment 1474nt							
ORF1 NA	47-1453; 467aa	neuraminidase [Influenza B virus (B/North Carolina/11/2017)] ARO42774.1; 91%; 43.09%	2×10^{-125}	Sialidase super family	cl21531	88–465	2.99×10^{-108}
ORF2 NB	52-366; 103aa						
M Segment 1194nt							
ORF1	29-793; 253aa	matrix protein 1 [Influenza A virus] QFX66948.1; 99% 41.50%	4×10^{-77}	Flu_M1<br>Flu_M1_C super family	pfam00598<br>cl27878	4–157<br>161–252	4.92×10^{-30}<br>9.38×10^{-19}
ORF2	793-1167; 123aa	BM2 protein [Influenza B virus] AAD29189.1; 82%; 30.19%	1.5	Flu_B_M2 super family	cl04739	8–108	7.07×10^{-4}
NS Segment Isoform 1: 1081nt							
ORF1	23-880; 285aa	NS [Wuhan spiny eel influenza virus] 13%; 51.28%	0.61	Flu_B_NS1 super family	cl03792	7–136	2.78×10^{-4}
ORF2	714-1058; 114aa	NEP [Influenza A virus (A/swine/Netherlands/Groenlo-37/2012(H1N1))] 100%; 24.56%	0.1	Influenza non-structural protein (NS2)	pfam00601	22–114	0.0017
NS Segment Isoform 2: 669nt, Splice junction 1–298:711–1081 of NS segment isoform 1 as reference							
ORF1	23-646 132aa	NEP [Influenza A virus (A/swine/Netherlands/Groenlo-37/2012(H1N1))] AKJ80762.1; 88%; 23.93%	0.089	Influenza non-structural protein (NS2)	pfam00601	40–132	0.0028

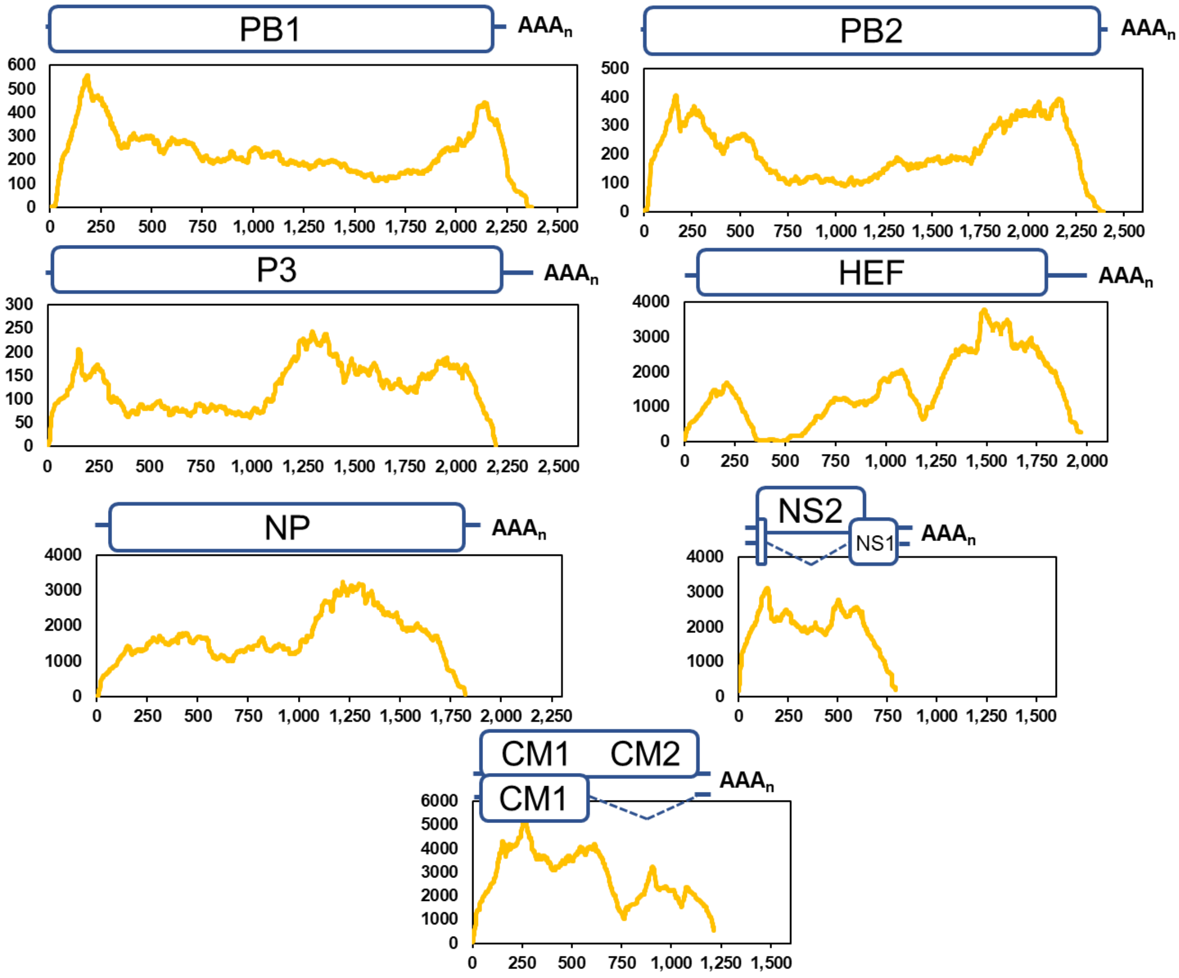

Figure S4. Genome organization and mapping coverage of Ornate chorus frog influenza-like virus. Coloured boxes correspond to predicted protein domains. PB1, RNA-dependent RNA polymerase basic subunit 1; PB2, RNA-dependent RNA polymerase basic subunit 2; PA, RNA-dependent RNA polymerase acidic subunit; NP, nucleoprotein; HEF, Hemagglutinin esterase; CM1/CM2, Influenza C virus matrix proteins 1 & 2; NS1/NS2, non-structural protein 1 & 2;. Isoform variants of genes indicated by dotted lines. Sequencing data taken from lung tissues of stage 28 tadpole (SRA accession: SRR5557878) [4].

Table S5. Annotation of Ornate chorus frog influenza-like virus.

	Location; Amino acid size	Closest BLASTp hit; Query cover (%); Identity (%)	*E*-value	Protein domain families	Accession	Interval	*E*-value
PB1 Segment 2375nt							
ORF1 PB1	14-2314; 765aa	polymerase PB1 [Influenza D virus (D/bovine/Mississippi/ C00046N/2014)] ALE66333.1; 98%; 81.91%	0	Flu_PB1	pfam0060	14–749	0
PB2 Segment 2395nt							
ORF1 PB2	29-2396; 712aa	polymerase PB2 [Influenza D virus (D/swine/Italy/173287-4/2016)] AON76692.1; 99; 68.22%	0	Flu_PB2 super family	cl20020	5–771	0
P3 Segment 2196nt							
ORF1 P3	26-2127; 712aa	polymerase 3 [Influenza D virus (D/bovine/ Shandong/Y127/2014)] AIE52107.1; 99%; 63.78%	0	Flu_PA super family	cl02905	20–705	0
HEF Segment 2263nt							
ORF1 HEF	124-2205; 693aa	hemagglutinin-esterase [Influenza C virus (C/Minnesota/25/2015)] AST08262.1; 92%; 40.95%	2×10^{-163}	Hema esterase super family	cl28016	39–417	4.45×10^{-66}
				Hema_stalk super family	cl07368	448–619	5.29×10^{-58}
NP Segment 1823nt							
ORF1 NP	33-1751; 571aa	nucleoprotein [Influenza D virus (D/bovine/ Yamagata/10710/2016)] BBC14931.1; 89%; 54.62%	0	Flu_NP super family	cl27387	3–498	9.48×10^{-24}
M Segment Isoform 1: 1273nt							
ORF1	63-1241; 392aa	P42 [Influenza D virus (D/bovine/Texas/3-13/2011)] AIO11642.1; 91%; 51.81%	2×10^{-128}	CM1 super family	cl03846	1–233	2.69×10^{-70}
				CM2 super family	cl03843	241–388	4.58×10^{-3}
M Segment Isoform 2: 980nt, Splice junction 1–791:1081–1273 of M segment isoform 1 as reference							
ORF1 CM1-CM2	63-803 246aa	P42 [Influenza D virus (D/bovine/Kansas/11-8/2012)] AIO11618.1; 99%; 59.27%	6×10^{-92}	CM1 super family	cl03846	1–233	2.69×10^{-70}
NS Segment Isoform 2: 867nt							
ORF1 CM1	36-740; 234aa	nonstructural protein 1 [Influenza D virus (D/bovine/Minnesota/729/2013)] AGS48800.1; 99; 48.74%	5×10^{-75}	Flu_C_NS1 super family	cl04118	82–205	2.50×10^{-9}
				Flu_C_NS2 super family	cl04153	7–63	2.56×10^{-5}
ORF2	483-791; 102aa	nonstructural protein 2 [Influenza D virus] BBM60900.1; 98%; 46.53%	4×10^{-23}				
NS Segment Isoform 2: 518nt, Splice junction 1–126:473–863 of NS segment isoform 1 as reference							
ORF1	36-491: 151aa	NS2 [Influenza D virus (D/bovine/Oklahoma/660/2013)] AGS48808.1; 100%; 39.13%	1×10^{-29}				

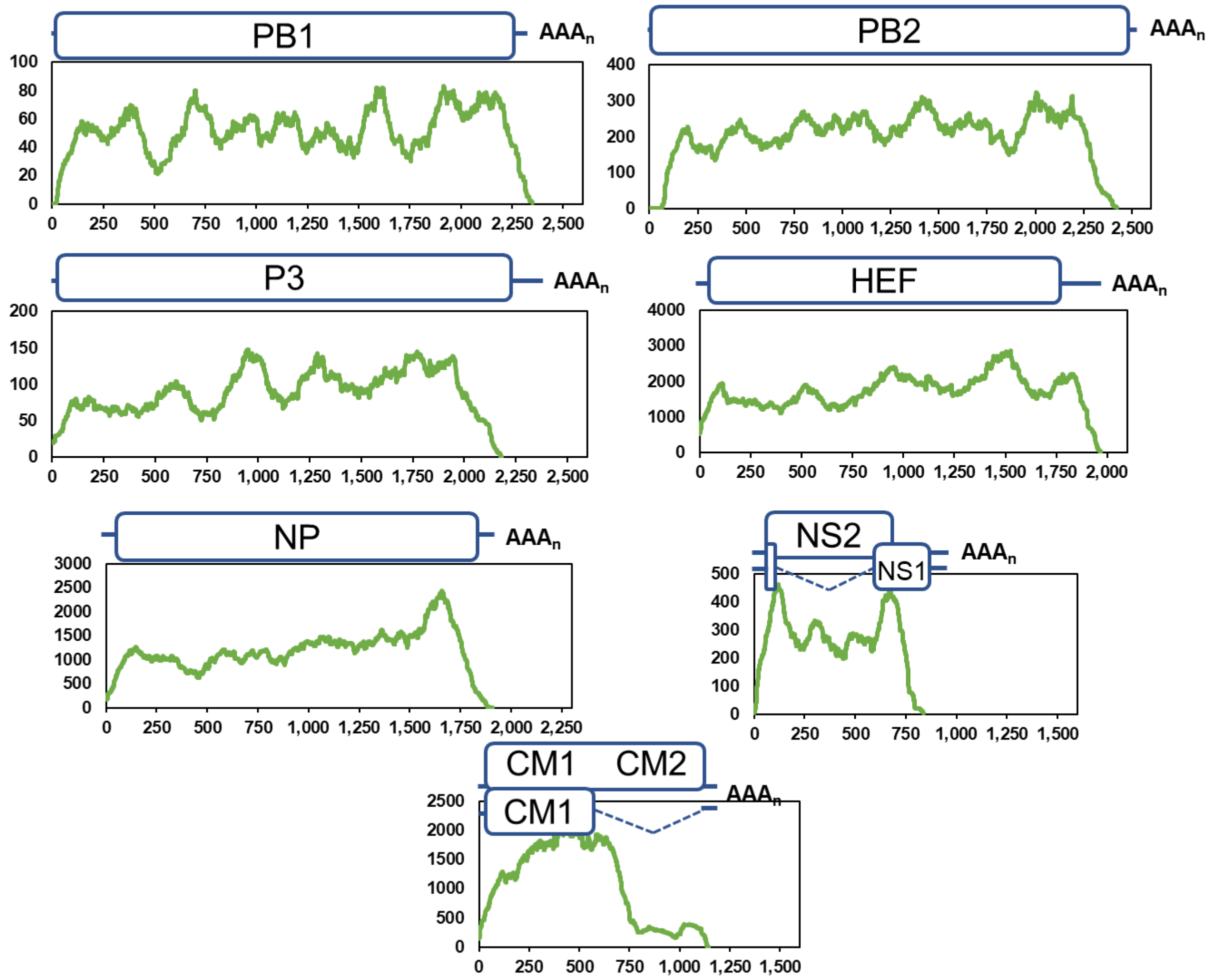

Figure S5. Genome organization and mapping coverage of Cane toad influenza-like virus. Coloured boxes correspond to predicted protein domains. PB1, RNA-dependent RNA polymerase basic subunit 1; PB2, RNA-dependent RNA polymerase basic subunit 2; PA, RNA-dependent RNA polymerase acidic subunit; NP, nucleoprotein; HEF, Hemagglutinin esterase; CM1/CM2, Influenza C virus matrix proteins 1 & 2; NS1/NS2, non-structural protein 1 & 2;. Isoform variants of genes indicated by dotted lines. Sequencing data taken from whole larval samples from Innisfail, QLD (SRA accessions: SRR5446725, SRR5446726) [5].

Table S6. Annotation of Cane toad influenza-like virus.

	Location; Amino acid size	Closest BLASTp hit; Query cover (%); Identity (%)	*E*-value	Protein domain families	Accession	Interval	*E*-value
PB1 Segment 2350nt							
ORF1 PB1	44-2308; 753aa	polymerase PB1 [Influenza D virus (D/bovine/Mexico/ S7/2015)] AMN87903.1; 99%; 77.03%	0	Flu_PB1	pfam0060	1–734	0
PB2 Segment 2421nt							
ORF1 PB2	86-2419; 777aa	polymerase PB2 [Influenza D virus (D/bovine/Kansas/14-22/2012)] AIO11621.1; 99%; 62.87%	0	Flu_PB2 super family	cl20020	5–774	0
P3 Segment 2180nt							
ORF1 P3	4-2151; 714aa	polymerase P3 [Influenza D virus (D/bovine/Yamagata/ 10710/2016)] BBC14929.1; 100%; 62.66%	0	Flu_PA super family	cl02905	24–707	0
HEF Segment 1968nt							
ORF1 HEF	6-1967; 653aa	hemagglutinin-esterase [Influenza D virus (D/bovine/Ibaraki/7768/2016)] BAV17997.1; 100%; 40.66%	5×10^{-169}	Hema esterase super family<br>Hema_stalk super family	cl28016<br>cl07368	62–417<br>449–620	1.24×10^{-60}<br>3.78×10^{-48}
NP Segment 1911nt							
ORF1 NP	15-1817; 599aa	nucleoprotein [Influenza D virus (D/bovine/Yamagata/ 10710/2016)] BBC14931.1; 87%; 49.34%	0	Flu_NP super family	cl27387	27–496	8.51×10^{-22}
M Segment Isoform 1: 1181nt							
ORF1 CM1-CM2	34-1167; 377aa	P42 [Influenza D virus (D/bovine/Texas/3-13/2011)] AIO11642.1; 99%; 46.15%	3×10^{-95}	CM1 super family<br>CM2 super family	cl03846<br>cl03843	1–233<br>236–370	1.17×10^{-67}<br>1.89×10^{-03}
M Segment Isoform 2: 967nt, Splice junction 1–761:1028–1233 of M segment isoform 1 as reference							
ORF1 CM1	34-768; 244aa	P42 [Influenza D virus (D/bovine/Texas/3-13/2011)] AIO11642.1; 99%; 58.44%;	3×10^{-85}	CM1 super family	cl03846	1–233	1.17×10^{-67}
NS Segment Isoform 1: 839nt							
ORF1	30-710; 226aa	NS11 [Influenza D virus (D/bovine/Shandong/Y127/2014)] AIE52112.1; 100%; 44.59%	3×10^{-64}	Flu_C_NS1 super family<br>Flu_C_NS2 super family	cl04118<br>cl04153	81–205<br>7–63	8.55×10^{-12}<br>3.79×10^{-11}
ORF2	508-837; 109aa	NS2 [Influenza D virus (D/bovine/ Oklahoma/660/2013)] AGS48808.1; 96%; 33.96%	0.079				
NS Segment Isoform 2: 595nt, Splice junction 1–225:476–845 of NS segment isoform 1 as reference							
ORF1	36-593; 185aa	NS2 [Influenza D virus (D/bovine/Mexico/S7/2015)] AMN87888.1; 100%; 42.16%	7×10^{-31}	Flu_C_NS2 super family	cl04153	7–63	3.79×10^{-11}

References

1. Sabin, K. Z.; Jiang, P.; Gearhart, M. D.; Stewart, R.; Echeverri, K., AP-1(cFos/JunB)/miR-200a regulate the pro-regenerative glial cell response during axolotl spinal cord regeneration. *Commun Biol* **2019,** *2*, 91.
2. Lee, S. Y.; Lee, H. J.; Kim, Y. K., Comparative transcriptome profiling of selected osmotic regulatory proteins in the gill during seawater acclimation of chum salmon (*Oncorhynchus keta*) fry. *Sci Rep* **2020,** *10*, 1987.
3. Sun, Y.; Huang, Y.; Li, X.; Baldwin, C. C.; Zhou, Z.; Yan, Z.; Crandall, K. A.; Zhang, Y.; Zhao, X.; Wang, M.; et al. Fish-T1K (Transcriptomes of 1,000 Fishes) Project: large-scale transcriptome data for fish evolution studies. *Gigascience* **2016,** *5*, 18.
4. Liu, L.; Wang, S.; Zhao, L.; Jiang, J., De novo transcriptome assembly for the lung of the ornamented pygmy frog (*Microhyla fissipes*). *Genom Data* **2017,** *13*, 44-45.
5. Edwards, R. J.; Tuipulotu, D. E.; Amos, T. G.; O'Meally, D.; Richardson, M. F.; Russell, T. L.; Vallinoto, M.; Carneiro, M.; Ferrand, N.; Wilkins, M. R.; et al. Draft genome assembly of the invasive cane toad, Rhinella marina. *Gigascience* **2018,** *7*, giy095.
